# Pathophysiologic and Transcriptomic Analyses of Viscerotropic Yellow Fever in a Rhesus Macaque Model

**DOI:** 10.1371/journal.pntd.0003295

**Published:** 2014-11-20

**Authors:** Flora Engelmann, Laurence Josset, Thomas Girke, Byung Park, Alex Barron, Jesse Dewane, Erika Hammarlund, Anne Lewis, Michael K. Axthelm, Mark K. Slifka, Ilhem Messaoudi

**Affiliations:** 1 Division of Biomedical Sciences, School of Medicine, University of California Riverside, Riverside, California, United States of America; 2 Laboratoire de Virologie Est, Centre de Biologie et de Pathologie Est, Hospices Civils de Lyon, Bron, France; 3 Department of Botany and Plant Sciences, University of California Riverside, Riverside, California, United States of America; 4 Public Health and Preventive Medicine, Oregon Health and Science University, Portland, Oregon, United States of America; 5 Vaccine and Gene Therapy Institute, Oregon Health and Science University, Portland, Oregon, United States of America; 6 Division of Pathobiology and Immunology, Oregon National Primate Research Center, Portland, Oregon, United States of America; 7 Division of Neuroscience, Oregon National Primate Research Center, Portland, Oregon, United States of America; 8 Division of Comparative Medicine, Oregon National Primate Research Center, Portland, Oregon, United States of America; University of Texas Medical Branch, United States of America

## Abstract

Infection with yellow fever virus (YFV), an explosively replicating flavivirus, results in viral hemorrhagic disease characterized by cardiovascular shock and multi-organ failure. Unvaccinated populations experience 20 to 50% fatality. Few studies have examined the pathophysiological changes that occur in humans during YFV infection due to the sporadic nature and remote locations of outbreaks. Rhesus macaques are highly susceptible to YFV infection, providing a robust animal model to investigate host-pathogen interactions. In this study, we characterized disease progression as well as alterations in immune system homeostasis, cytokine production and gene expression in rhesus macaques infected with the virulent YFV strain DakH1279 (YFV-DakH1279). Following infection, YFV-DakH1279 replicated to high titers resulting in viscerotropic disease with ∼72% mortality. Data presented in this manuscript demonstrate for the first time that lethal YFV infection results in profound lymphopenia that precedes the hallmark changes in liver enzymes and that although tissue damage was noted in liver, kidneys, and lymphoid tissues, viral antigen was only detected in the liver. These observations suggest that additional tissue damage could be due to indirect effects of viral replication. Indeed, circulating levels of several cytokines peaked shortly before euthanasia. Our study also includes the first description of YFV-DakH1279-induced changes in gene expression within peripheral blood mononuclear cells 3 days post-infection prior to any clinical signs. These data show that infection with wild type YFV-DakH1279 or live-attenuated vaccine strain YFV-17D, resulted in 765 and 46 differentially expressed genes (DEGs), respectively. DEGs detected after YFV-17D infection were mostly associated with innate immunity, whereas YFV-DakH1279 infection resulted in dysregulation of genes associated with the development of immune response, ion metabolism, and apoptosis. Therefore, WT-YFV infection is associated with significant changes in gene expression that are detectable before the onset of clinical symptoms and may influence disease progression and outcome of infection.

## Author Summary

Yellow fever virus causes ∼200,000 infections and 30,000 deaths annually in Africa and South America. Although this is an important human pathogen, the basis of yellow fever disease severity remains poorly understood. Rhesus macaques are susceptible to yellow fever and develop similar symptoms as severe as those observed in humans. In this study, we characterized disease progression in this model and observed a profound loss of lymphocytes that preceded the appearance of serum markers of virus-induced liver pathology. This change might provide an early indicator of fatal yellow fever. In addition, we also identified significant changes in gene expression in white blood cells that occur before any measurable disease symptoms and these genetic signatures may provide future targets for antiviral therapeutics and better diagnostics.

## Introduction

Yellow fever virus (YFV) is a member of the flavivirus genus and is endemic or intermittently epidemic in 45 countries (32 in Africa and 13 in South America) [1], [2]. YFV causes ∼200,000 cases and 30,000 deaths annually [3]. There are two main life cycles for YFV: in the urban cycle, YFV is transmitted between humans primarily through the bite of infected *Aedes aegypti* mosquitoes; in the jungle cycle, YFV is transmitted between nonhuman primates via *Hemagogus* mosquitoes in South America and *Aedes africanus* in Africa [4].

The clinical symptoms of yellow fever (YF) can be quite broad, ranging from mild disease to severe manifestations including liver and kidney failure and hemorrhage [3], [5]. YF is characterized by three stages. During the incubation period, which lasts 3–4 days, virus can be detected in the blood and patients may experience fever, myalgia, and nausea. This is usually followed by remission with abatement of symptoms for 24–48 hours. In some patients, this is followed by the return of symptoms at a more severe level and the onset of jaundice. Deepening jaundice, rising pulse, hypotension, and hypothermia, appear before death, which occurs in 20 to 50% of cases [6]. The current live attenuated YFV vaccines are effective, but are not without complications [7], [8]. YF vaccine-associated neurotropic disease (YEL-AND) and YF vaccine-associated viscerotropic disease (YEL-AVD) are rare but represent serious adverse events [7], [9]–[13]. YF vaccines are contraindicated in infants <9 months of age, people with primary immunodeficiencies, malignant neoplasms, organ transplant, AIDS or other clinical manifestations of HIV, and thymus disorders (thymoma, myasthenia gravis, or thymic ablation) [14], [15]. Therefore the development of a safer vaccine is highly desirable [16]. In addition, as with all pathogens, increased travel increases the risk of outbreaks in areas with high-density vectors and an unvaccinated population. These concerns are further compounded by the lack of approved antivirals for YF. In order to develop new vaccine and therapeutic strategies, we need a better understanding of the pathophysiology of YF.

Small animal models of YFV infection such as Golden hamsters [17]–[19] or mice that are genetically deficient in IFNαβγ receptor expression (AG129 mice) [20] have been developed. Although these small animal models offer several advantages, they also have caveats. For example, the hamster model requires the use of a hamster-adapted strain of YFV (YFV-Jimenez), and unfortunately many immunological reagents are not readily available for this species. Infection of the AG129 mouse model with the vaccine strain of YFV-17D results in lethal viral encephalitis but not viscerotropic disease [20]. More importantly, the host immune response to YFV infection cannot be adequately studied in an immune deficient host such as AG129 mice. In contrast, non-human primates (NHP) provide a very robust model for studying YFV since these animals represent a natural reservoir during the jungle cycle of transmission [15] and the clinical manifestations following lethal YFV challenge of rhesus macaques closely mimic severe forms of human viscerotropic disease [21]. Indeed, large YF outbreaks in NHP populations have been reported in areas where human epizootics have occurred. For instance, between October 2008 and June 2009, over 2000 howler monkeys succumbed to YF in Brazil during the same time as 21 confirmed human cases [22].

In this study, we characterized viral dissemination; changes in immune cell frequencies both in peripheral blood and lymphoid tissues; as well as changes in cytokine and liver enzyme levels in NHP infected with YFV-DakH1279. Data presented herein show that a profound loss of peripheral lymphocytes in the blood precedes characteristic liver pathology and provides an early indicator of fatal YF in this model. In addition, we examined the transcriptome in peripheral blood mononuclear cells (PBMC) collected 3 days after infection with YFV-DakH1279 compared with the attenuated vaccine strain, YFV-17D. This analysis revealed that striking changes in gene expression are evident at this early time point and provide glimpses into the molecular basis of YFV virulence.

## Methods and Materials

### Virus

YFV-DakH1279 (originally isolated from a YF patient in Senegal in 1965) was obtained from the World Reference Center for Emerging Viruses and Arboviruses after approval from Dr. Robert Tesh (University of Texas Medical Branch, Galveston, TX). The initial inoculum was passaged once in a young rhesus macaque (∼10^3^ TCID_50_) and the animal developed viscerotropic disease and required humane euthanasia at 5 days post infection. Serum from the YFV-DakH1279 infected macaque collected at necropsy was then passaged once on C6/36 cells grown in EMEM supplemented with 10% FBS and antibiotics at 28°C, 6% CO_2_ to prepare a low-passage virus stock for *in vivo* pathogenesis studies at a titer of 9.4×10^5^ infectious units/mL. Since YFV-DakH1279 does not form plaques, cytopathic effect (CPE), or measurable focus forming units, we used a flow cytometry-based tissue culture limiting dilution assay (TC-LDA) to determine the infectious virus titer. The TC-LDA [functionally similar to a tissue culture infectious dose-50 (TCID_50_)] was performed by incubating serial dilutions of virus in replicate wells of C6/36 mosquito cells and stained intracellularly with a YFV-specific monoclonal antibody, 3A8.B6 as previously described [23].

### YFV quantitative real-time PCR

RNA isolation was performed using the ZR Viral RNA kit per the manufacturer's instructions (Zymo Research). Briefly, 200 µL of serum was transferred to a tube containing 30 µL of ZR Viral RNA Buffer. This mixture was bound to a Zymo-Spin IC Column by centrifugation at 16,000× g for 2 minutes. The flow-through was discarded, and the column was washed twice with 300 µL of RNA Wash Buffer. Residual wash buffer was removed by centrifugation, and the purified RNA was eluted with 12 µL of RNase-free water.

Purified RNA was reverse transcribed using the High Capacity cDNA Reverse Transcription Kit (Applied Biosystems) following the manufacturer's instructions for 20 µL reactions. YFV genome copy numbers were then measured by quantitative PCR (qPCR) using the following forward (5′CAC GGA TGT GAC AGA CTG AAG A 3′) and reverse (5′CCA GGC CGA ACC TGT CAT 3′) primers and probe (5′ 6-FAM- CGACTGTGTGGTCCGGCCCATC 3′BHQ). Standard curve was established using the following amplicon as template (CGA CTG TGT GGT CCG GCC CAT CCA CGG ATG TGA CAG ACT GAA GAG GAT GGC GGT GAG TGG AGA CGA CTG TGT GGT CCG GCC CAT CGA TGA CAG GTT CGG CCT GG). In these experiments, cDNA was subjected to 10 min@95°C followed by 40 cycles of [15 sec@95°C/1 min@60°C]. Experiments were carried out using a StepOnePlus Real-Time PCR system (Applied Biosystems). Viral load in a subset of serum samples were also measured using the TC-LDA method described above [23].

### Ethics statement

All Rhesus macaques were handled in strict accordance with the recommendations described in the Guide for the Care and Use of Laboratory Animals of the National Institute of Health, the Office of Animal Welfare and the United States Department of Agriculture. All animal work was approved by the Oregon National Primate Research Center (ONPRC) Institutional Animal Care and Use Committee (PHS/OLAW Animal Welfare Assurance # A3304-01). The ONPRC is fully accredited by the Assessment and Accreditation of Laboratory Animal Care-International. Animals were housed in adjoining individual primate cages allowing social interactions, under controlled conditions of humidity, temperature and light (12-hour light/12-hour dark cycles). Food (commercial monkey chow supplemented by treats and fruit twice daily) and water were available ad libitum. Environmental enrichment consisted of commercial toys. All procedures were carried out under Ketamine anesthesia by trained personnel under the supervision of veterinary staff and all efforts were made to minimize animal suffering. After infection, trained personnel monitored animals 4 times a day. Monkeys were humanely euthanized by the veterinary staff at ONPRC in accordance with endpoint policies. Euthanasia was conducted under anesthesia with ketamine followed by overdose with sodium pentobarbital. This method is consistent with the recommendation of the American Veterinary Medical Association.

### Animal studies and sample collection

Twenty female rhesus macaques (*Macaca mulatta*) 8–16 years of age were used in these studies. Animals were assigned to Animal Biosafety Level-3 (ABSL-3) housing in successive cohorts ranging from 2 to 4 animals and infected subcutaneously with YFV-DakH1279 at doses ranging from 25 to 5×10^4^ infectious units (n = 2–4/dose). Blood samples were collected on days 0, 3, 4, 5, 6, 7, 10, and 14 post-infection. Complete blood counts and liver enzymes were determined every time a blood sample was collected. Animals were euthanized if 4 out the 6 criteria listed below were reached: 1) >80% decrease in number of circulating lymphocytes; 2) ALT levels >1000 U (normal <100 U); 3) bile acid (BA) levels >100 U (normal <10); 4) total bilirubin (TBIL) >1.5 mg/dl (normal <0.5 mg/dl); 5) weight loss >30%; and 6) viral loads >10^7^ genomes/ml serum. We used the cohort infected with the 5×10^4^ TCID50 (i.e., the first cohort) to develop the humane endpoints listed above. In this first challenge experiment, one of the animals euthanized presented only with high viral loads and lymphopenia, while blood chemistry profiles were within normal ranges. Following necropsy, the histopathology analysis showed minimal organ damage (Table 1), which suggested that this animal might have survived the challenge. Based on those observations, we made the decision to require humane euthanasia when 4 out of the 6 criteria were met. In subsequent experiments, two animals that did not meet the humane euthanasia endpoints were necropsied 7 and 10 dpi. The truncation of the study time course in the case of these two animals was due to Institutional Animal Care and Use Committee policy that requires animals be housed no longer than 24 hours without a companion animal in the room. This would have required additional animals to be assigned to ABSL-3 and the euthanasia of these additional animals. For humane reasons it was decided therefore, to necropsy the experimental animals before the full 14 days. At the time of necropsy, blood, liver, kidney, spleen, bone marrow, and axillary, inguinal and mesenteric lymph nodes were harvested from all animals.

**Table 1:** Summary of histological analysis of liver, lymphoid tissue, and kidney sections.

Dose	DPI	Hepatocellular Damage	Liver	Spleen	Lymph Node	Kidney
5×10^4^	5	81%	+++++ H, L	+++++	++	++++
	4	78%	+++++ H, L	++++	++	++++
	5	70%	++++ H, L	+++	++	++++
	5	<1%	+ V, L	+, M	+ M	none
10^4^	5	68%	++++ L	++	+	++++
	7	<1%	+ V, L	+, M	+ M	+
10^3^	5	77%	++++ L	+++	+	++++
	5	75%	++++ L	+++	++ M	+++
	5	63%	+++ L	+, M	+ M	+
	4	46%	++ L	+, M	+ M	+
	4	33%	++	M	M	R
	10	<1%	+, V, L	+, M	+	none
10^2^	6	57%	+++ L	+, M	M	+
	5	38%	++	+, M	+ M	+
	4	36%	++	M	none	+
	14	<1%	+ V, L	+, M	+ M	none
25	6	79%	+++++ L	++++	+ M+	++++
	7	66%	+++ L	+, M	+ M	+
	7	39%	+++	+, M	+ M	+
	14	0%	R	+, M	M	none

Liver + = mid-zonal necrosis severity; H = hemorrhage; L = lymphatic infiltration; V = vacuolization; R = regeneration; Spleen & Lymph Node + = apoptosis severity; M = mitosis; Kidney + = granular and proteinaceous cast severity.

Three additional animals were infected with 1 standard dose (0.5 ml) of YFV-17D (YF-Vax, Sanofi Pasteur, formulated to contain no less than 4.74 log_10_ PFU/0.5 ml) subcutaneously. Blood samples were collected prior to and 3 days post-infection for gene expression analysis.

### Liver and hematological analyses

Total white blood cell count, lymphocyte, platelet, red blood cell counts, hemoglobin, and hematocrit values, were determined from EDTA blood with the HemaVet 950FS+ laser-based hematology analyzer (Drew Scientific, Waterbury, CT).

Serum was analyzed for alkaline phosphatase (ALP), alanine aminotransferase (ALT), gamma glutamyltransferase (GGT), bile acid (BA), total bilirubin (TBIL), albumin (ALB), and blood urea nitrogen (BUN) using a VetScan VS2 (Abaxis veterinary diagnostics, Union City, CA).

### Measuring frequency of immune cell subsets

PBMC were surface stained with antibodies against CD8β (Beckman Coulter, Brea, CA), CD4 (eBioscience, San Diego, CA), CD20 (Beckman Coulter, Brea, CA), HLA-DR (eBioscience), and CD14 (Biolegend, San Diego, CA). Samples were fixed with 4% paraformaldehyde for 4 hrs before removal from the BSL-3. The samples were acquired using the LSRII instrument (Beckton Dickenson, San Jose, CA) and the data were analyzed using FlowJo software (TreeStar, Ashland, OR).

### Plasma cytokine levels

Aliquots of plasma samples (stored at −80°C) were thawed and heat inactivated for 60 min at 55°C for removal from the BSL-3. Heat inactivated serum samples must be tested for residual live virus before removal from the BSL-3. Samples were then analyzed with Milliplex Non-Human Primate Magnetic Bead Panel containing the following analytes: TNFα, IL-6, IL-12/23p40, IL-8, MCP-1, IL1Ra, soluble CD40L, IL-15, IFNγ, IL-4 and IL-17 as per manufacturer's instructions (Millipore Corporation, Billerica, MA). Heat inactivation decreased the detection of the cytokines in this kit as follows: TNFα by 42%, IL-6 by 53%, IL-12/23p40 by 56%, IL-8 by 20%, MCP-1 by 33%, IL1Ra by 96%, IL-15 by 49%, IFNγ by 73%, and IL-17 by 19%. We were unable to determine the impact of heat inactivation on the levels of CD40L and IL-4 since the levels of these analytes were below detection in the test samples we subjected to this treatment.

### Immunohistochemistry

Tissues were collected and placed in neutral-buffered formalin for paraffin embedding. Sections were cut at 5 µm, deparaffinized and stained with hematoxylin and eosin, or blocked with 5% normal goat serum and 5% bovine serum albumin for immunostaining with primary antibodies specific for YFV antigen (mouse anti-YF clone 3A8.B6; 1.5 µg/µL, a generous gift from Dr. Ian Amanna), B cells (mouse anti-human CD20, Dako; 1∶475), or T cells (rabbit anti-human CD3, Dako; 1∶200). Secondary antibodies used were: biotinylated goat-anti-mouse IgG and biotinylated goat-anti-rabbit IgG (Vector; 1∶300). DAB chromagen with hematoxylin counterstain (Vector) was used to visualize CD20+ B cells and CD3+ T cells. VIP substrate with methyl green counterstain (Vector) was used to visualize YFV antigen. The sections were then analyzed and images captured using an Axioplan microscope (Carl Zeiss) with a Spot Insight camera (Diagnostic Insturments Inc.)

### Gene expression

Microarray assays were performed in the OHSU Gene Profiling Shared Resource. One million PBMC were resuspended in Trizol (Invitrogen) and RNA was extracted using RNeasy Micro Kit (Qiagen) according to the manufacturer's protocol. Total RNA was treated with RNase-free DNase (Qiagen) followed by purification and concentration with the RNA Clean & Concentrator-5 kit (Zymo Research). Following clean-up, 25 ng of total RNA from each sample were amplified and biotin-labeled using the Ovation RNA Amplification System V2, Ovation WB Reagent, and Encore Biotin Module (NuGEN Technologies) as per manufacturer recommendations. Labeled hybridization targets were mixed with hybridization solution containing hybridization controls (Affymetrix) according to NuGEN Technologies protocol and hybridized with the GeneChip Rhesus Macaque Genome Array (Affymetrix). This array contains 52,024 probe sets interrogating over 47,000 *M. mulatta* transcripts. Arrays were scanned using the GeneChip Scanner 3000 7G and image quality was determined immediately following each scan. Image processing was performed with Affymetrix GeneChip Command Console v3.1.1 and probe set summarization and CHP file generation were performed using Affymetrix Expression Console v1.1 software.

### Microarray data analysis

All microarray data analysis steps were performed in the statistical environment R, using Bioconductor packages (R Development Core Team, 2008). The probe set-to-gene mappings for the Rhesus chip were downloaded from the Affymetrix site. All ambiguous probe sets on this chip were treated in the gene enumeration steps of this study in the following manner: controls and probe sets matching no or several loci in the *Macaca mulatta* genome were ignored in the downstream analysis steps. In addition, redundant probe sets that represent the same locus several times were counted only once. The normalization of the raw data CEL files was performed with the Robust Multi-array Average (RMA) algorithm using the default settings of the corresponding R function [24]. The quality of the Affymetrix Gene Chips was assessed with analysis routines provided by the affyPLM library [25]. For each probe, log_2_ fold change (log_2_FC) expression was calculated as the difference of log_2_ expression at 3 days post-infection (dpi) relative to 0 dpi. Analysis of differentially expressed genes (DEGs) was performed with the LIMMA package using the normalized expression values [26]. The Benjamini and Hochberg method was selected to adjust p-values for multiple testing and control false discovery rates (FDRs) [27]. As confidence threshold for identifying DEGs we chose an adjusted p-value of < = 0.05 and absolute log_2_FC superior to 1.

### Functional enrichment and upstream regulator analysis

Functional analysis of statistically significant gene expression changes was performed using Ingenuity Pathways Knowledge Base (IPA; Ingenuity Systems) and Gene Ontology (GO) [28]. In addition, we also used previously published microarray data from resting and activated human immune cells (GSE22886; IRIS database) to define genes specific to each immune cell type as previously described [29]. Genes specific to innate immune cells were further defined as the union of genes significantly up-regulated in resting or activated dendritic cells, natural killer cells, monocytes or neutrophils. Genes specific to adaptive immune cells were defined as the union of genes significantly up-regulated in naïve or activated T or B cells. For all gene set enrichment analyses, a right-tailed Fisher's exact test was used to calculate a p-value determining the probability that each biological function assigned to that data set was due to chance alone. An enrichment score (ES), defined as −log10 (p-value) as calculated using a right tailed Fisher's exact test, was calculated. In addition, we used the IPA regulation z-score algorithm which identifies biological functions that are expected to be activated or inhibited in infected animals *vs.* controls, and which is designed to reduce the chance that random data will generate significant predictions. Z-scores ≥2, indicate that the function is significantly increased and z-scores ≤−2, indicate that the function is significantly decreased.

### Publicly available data

Raw microarray data have been deposited in NCBI's Gene Expression Omnibus and are accessible through GEO series accession number GSE51972.

## Results

### YFV-DakH1279 replicates to high systemic levels

**Figure 1:**
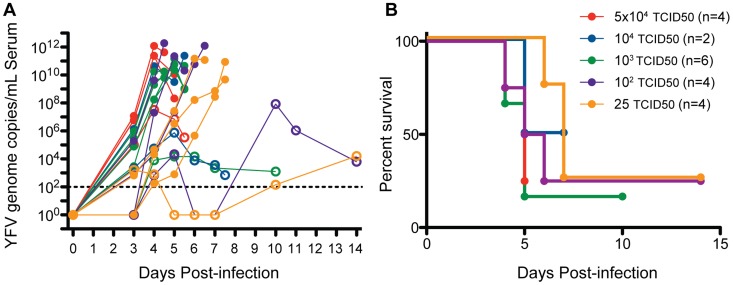
Wild type yellow fever virus (YFV-DakH1279) is highly virulent in rhesus macaques. Twenty adult female rhesus macaque (8–16 years) were infected subcutaneously with YFV-DakH1279 strain with doses ranging from 25 to 5×10^4^ infectious units. (A) Viral loads were determined using quantitative RT-PCR and are expressed as genome copy number/ml serum. Filled circles denote animals that required humane euthanasia and open circles denote animals that survived YFV infection. (B) Kaplan-Meier survival curves following YFV-DakH1279 infection.

Infection of rhesus macaques with 25 TCID_50_ to 5×10^4^ TCID_50_ of YFV-DakH1279 resulted in a fulminating disease that typically lasted 4–7 days (Figure 1). Higher doses of YFV-DakH1279 resulted in slightly higher and earlier viremia than lower doses of virus (Figure 1A). Peak viremia occurred between days 3 and 7 post-infection and in lethal cases reached 10^9^ to 10^13^ YFV genome equivalents/mL as measured by qRT-PCR shortly before the animals required humane euthanasia. We have previously showed a 1∶1 relationship between the levels of virus measured by qRT-PCR and the levels of infectious virus ([23], R^2^ = 0.89, p<0.0001), indicating that the YFV genome equivalents shown here are representative of the levels of infectious virus in circulation. Overall, we observed 75% lethality at 25 and 100 TCID_50_ (3/4 animals in each group) by 5–7 days post-infection (dpi); 84% lethality at 10^3^ TCID_50_ (5/6 animals) by 4–6 dpi; 50% mortality at 10^4^ infectious units (1/2 animals); and 75% lethality at a dose of 5×10^4^ TCID_50_ by 4–5 dpi (3/4 animals) (Figure 1B). All animals that controlled viral replication to below 10^6^ genome equivalents/mL during the first week of infection survived. Interestingly, two animals that survived at least 14 days after infection had received the lower challenge doses of virus (25 or 100 infectious units/animal) but presented with two successive rounds of viremia that occurred at 3–5 dpi and then again at 10–14 dpi.

### YFV-DakH1279 infection results in severe viscerotropic disease

**Figure 2:**
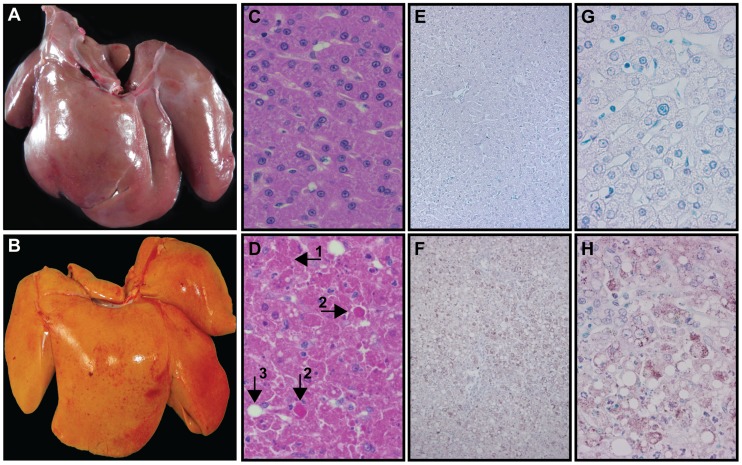
YFV-DakH1279 infection results in severe liver damage in rhesus macaques. (A–B) Images of liver in a representative uninfected (A) and YFV-DakH1279 infected animal that required humane euthanasia (B). The infected liver is discolored with signs of hemorrhagic foci. (C–D, 400×) H&E staining of liver sections from an uninfected (C) and YFV-DakH1279-infected (D) animal. Extensive hepatocytes necrosis (1) along with eosinophilic degeneration of liver cells (Councilman bodies, 2), and fatty changes (3) are noted by the black arrows in panel D. (E–H) Histological analysis of YFV antigen in an animal that survived (E 100× & G 400×) or required humane euthanasia following YFV-DakH1279 infection (F 100× &H 400×).

**Figure 3:**
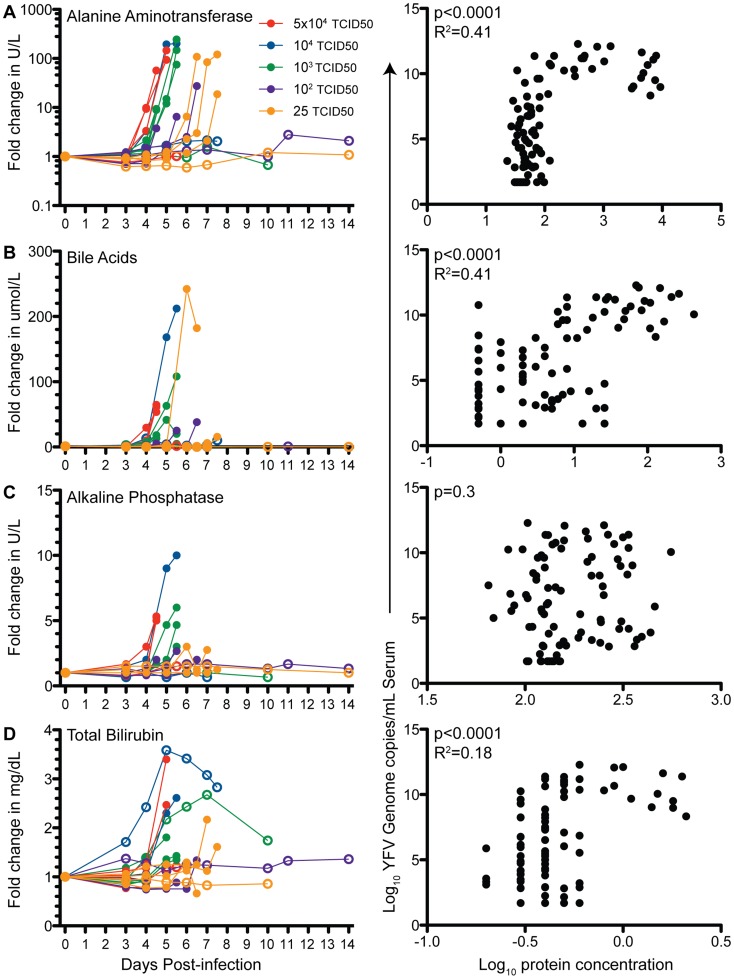
Indices of liver injury in YFV-DakH1279-infected rhesus macaques. Fold change in serum levels and correlation with viral genome copy number of alanine aminotransferase (A), bile acids (B), alkaline phosphatase (C) and total bilirubin (D) were determined at the indicated time points after infection. Filled circles denote animals that required humane euthanasia and open circles denote animals that survived YFV-DakH1279 infection.

In line with previous observations [21], animals that required humane euthanasia exhibited signs of significant liver injury. Unlike healthy liver from uninfected animals (Figure 2A), the livers of the animals that required euthanasia were discolored and contained hemorrhagic foci (Figure 2B). Histological examination revealed wide spread hepatocyte degeneration and necrosis, vacuolation and fatty changes (increased prevalence of lipid droplets) (Figure 2D, Table 1) that were not present in healthy liver (Figure 2C). We also detected councilman bodies, the hallmark of YF disease in the liver and extensive hemorrhage throughout the livers (Figure 2D). On rare occasions, we observed eosinophilic intranuclear inclusions (Torres bodies). YFV antigen was detected by immunohistochemistry in all liver sections obtained at necropsy from animals that required humane euthanasia (Figure 2F and 2H). In contrast, liver sections obtained from animals that survived YFV infection showed no evidence of YFV antigen (Figure 2E and 2G). In addition to these histological changes, we found sharp increases in serum levels of alanine aminotransferase (ALT), bile acids (BA), total bilirubin (TBIL) and alkaline phosphatasase (ALP) within 6–8 hours before the animals were euthanized (Figure 3). Levels of ALT reached 2000–9000 U/L in the most severe cases (normal <100 U/L) (Figure 3A); BA levels reached ∼100 umol/L (normal <10 umol/L) (Figure 3B); TBIL reached 1–1.5 mg/dl (normal <0.5 mg/dl) (Figure 3D). Changes in ALP on the other hand were less pronounced (Figure 3C) with one animal reaching 580 U/L (normal <200 U/L). In line with these observations, ALT, TBIL and BA showed significant curvilinear correlation with viral load (p<0.0001), whereas ALP levels showed no correlation (p = 0.3, Figure 3). Animals that survived infection exhibited little or no change in plasma levels of these key liver enzymes (Figure 3).

**Figure 4:**
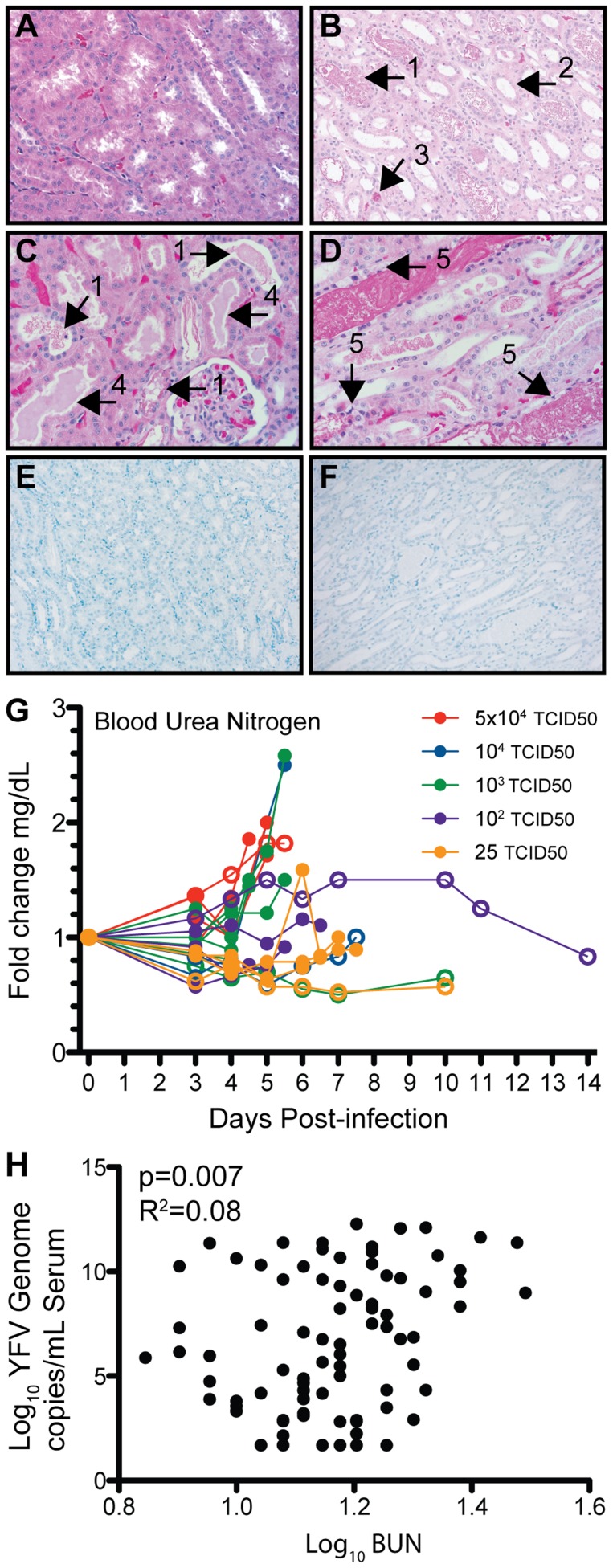
Kidney injury in YFV-DakH1279-infected rhesus macaques. (A–D) H&E staining of kidney sections from a representative uninfected (A) and YFV-DakH1279 infected animal (B–D). The black arrows note granular bilirubin casts in dilated distal convoluted tubules (1), necrotic tubular epithelial cells (2) and red blood cells (3), proteinaceous casts in dilated proximal convoluted tubules (4), and necrotic tubular epithelial cells in distal tubular segments containing large granular bilirubin casts (5). Histological staining of YFV antigen in kidney section from a surviving animal (E) and one that required euthanasia following YFV-DakH1279 infection (F) shows no detectable viral antigen. Slides A, C, and D acquired at 400× and slides B, E, and F acquired at 200× magnification. (G) Increased BUN levels were observed in some animals shortly before requiring euthanasia and (H) correlate with viral genome copy numbers/mL serum. Filled circles denote animals that required euthanasia and open circles denote animals that survived YFV infection.

Our analysis also revealed, that in contrast to kidney from uninfected animals (Figure 4A), evidence of kidney injury as indicated by renal tubular degeneration and epithelial tubular necrosis (Figure 4B, Table 1). We also detected granular bilirubin casts in dilated distal convoluted tubules and proteinaceous casts in kidneys from animals that required humane euthanasia (Figure 4B–D). Interestingly, YFV antigen was not detected in kidney tissue sections (Figure 4E–F), indicating that this is not a major site of active viral replication. Kidney dysfunction at the higher challenge doses was also indicated by a rise in blood urea nitrogen (BUN), averaging 1.5 and 1.9 -fold increase from baseline at TCID50 10^4^ and 5×10^4^). There was a significant correlation between challenge dose and fold changes in BUN (R2 = 0.97, p = 0.002). There was also a curvilinear correlation between BUN values and viral load (p = 0.007) with changes in BUN observed only approximately 6–8 hours before euthanasia (Figure 4G, F).

### YFV-DakH1279 infection causes severe lymphopenia

**Figure 5:**
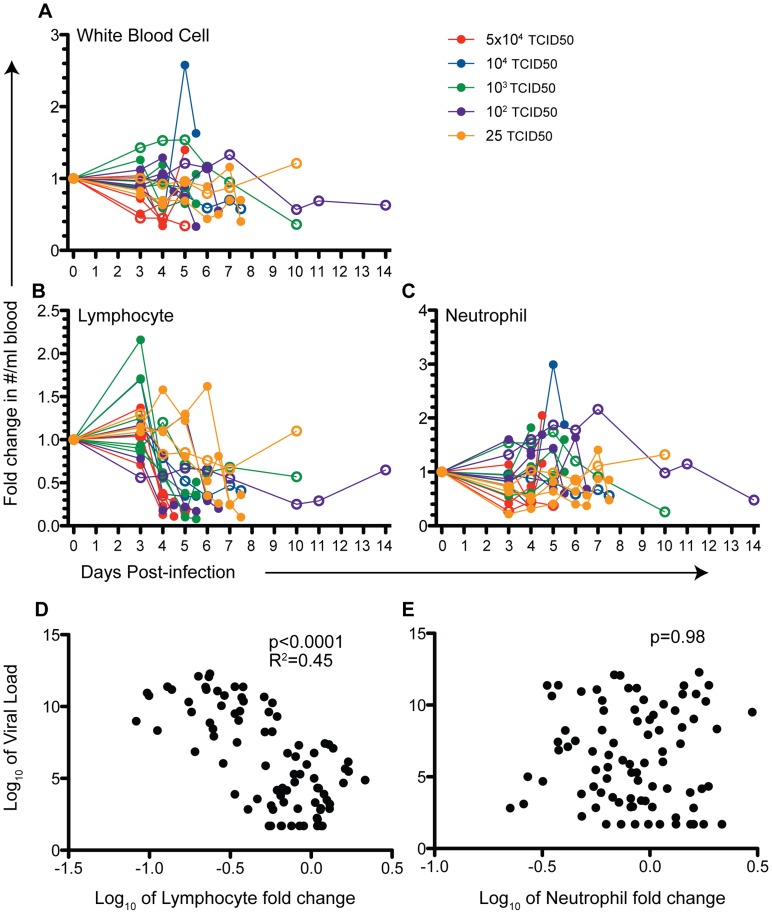
YFV-DakH1279 infection results in severe lymphopenia. (A) Fold change in white blood cells, (B) lymphocytes and (C) neutrophils were measured at the indicated time points and calculated as the ratio of cell counts/mL for each day relative to the 0 dpi count. (D) A negative correlation between viral load and lymphocyte count (p<0.0001, R^2^ = 0.46) (D), but no correlation was noted between numbers of circulating neutrophils and viral loads (E). Filled circles denote animals that required euthanasia and open circles denote animals that survived YFV infection.

We monitored changes in hematological parameters (Figure S1) and circulating white blood cells (WBC) throughout infection (Figure 5, Figure S2). Hematocrits, hemoglobin levels and platelet counts were stable until a few hours before the animals required humane euthanasia when small but significant decreases in platelet counts (p<0.01) and hematocrit (p<0.01) levels and a trend towards reduced hemoglobin levels (p = 0.07) were detected (Figure S1). We observed a modest decrease in total WBC counts between days 4–6 post-infection in animals with a lethal infection that required humane euthanasia (Figure 5A). This decrease was most evident in animals with the highest viral loads. In contrast, we found a severe loss of circulating lymphocytes, which declined by 71%±29.5 in animals that required euthanasia compared to a 23%±15.4 decline in animals that survived challenge (Figure 5B). Neutrophils declined at 3 dpi in most animals but increased slightly in others, resulting in an irregular pattern following YFV infection (Figure 5C). Indeed, we detected a significant negative correlation between viral load and extent of lymphocyte loss (R^2^ = 0.46, p<0.0001; Figure 5D) whereas no correlation between neutrophils and viral load was noted (Figure 5E). We further characterized the loss in lymphocyte subsets by measuring changes in both frequency and absolute numbers of CD4+ and CD8+ T cells, and CD20+ B cells (Figure S2). Frequencies of peripheral CD20+ B cells, CD4+ T cells and CD8+ T cells rapidly decreased, reaching nadir levels by about day 4 post-infection in the animals that ultimately required euthanasia (Figure S2 A–C). In the four animals that survived, frequencies of CD20+ B cells, CD4+ T cells and CD8+ T cells also declined but to a lesser extent and in two of the animals recovered to pre-infection levels days 10–14 post-infection (Figure S2 A–C). Numbers of circulating CD14+ monocytes in peripheral blood also decreased within 24 hours before the animals were euthanized (Figure S2). *In vitro* studies were performed to determine if YFV-17D or YFV-DakH1279 replicate in rhesus PBMC but no reproducible viral replication of either strain of virus in primary PBMC was found, indicating that it is unlikely that lymphocyte loss was due to direct viral infection.

**Figure 6:**
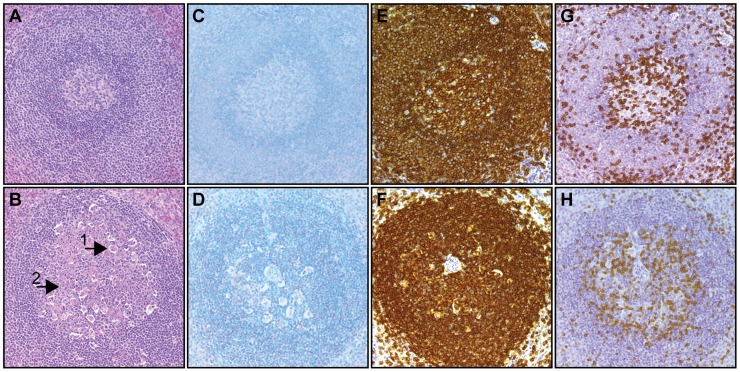
Germinal center necrosis following infection with YFV-DakH1279. (A, B) H&E staining showing healthy lymphocytic elements in the germinal centers of spleens from a surviving animal (A) and germinal necrosis in spleen of one that required euthanasia (B) Tangible body macrophage (1) and apoptosis (2) are indicated in panel B. (C, D) Histological examination of YFV antigen in spleen of a surviving animal (C) and one requiring euthanasia (D) show no viral antigen. (E–H) Histological staining for CD20 (E-survivor, F-euthanized) and CD3 antigens (G-survivor, H-euthanized). All slides were acquired at 200× magnification.

To further investigate the virus-induced lymphopenia, we examined lymphoid tissue collected from animals that survived and those with a lethal infection requiring humane euthanasia (Figure 6). Histological analysis showed that, in contrast normal cellular turnover observed in germinal center (GC) in spleen and lymph nodes in surviving YFV-DakH1279 infected animals (Figure 6A, Table 1), significant GC necrosis as indicated by increased apoptotic bodies and numerous tangible body macrophages was observed in animals that required euthanasia (Figure 6B; Table 1). Severity of GC necrosis correlated with infectious dose and was primarily observed in animals infected with 5×10^4^ and 10^4^ (Table 1). Moreover, several of the spleens examined were congested with evidence of hemorrhage. As described for the kidneys, we did not detect viral antigen in lymphoid tissue despite the GC necrosis (Figure 6C, 6D). We also examined distribution of CD20+ B cells and CD3+ T cells by immuno-histochemistry (IHC) in the spleen (Figure 6E–H). This analysis showed decreased B cell staining in the germinal center in animals that required euthanasia (Figure 6F).

### Cytokine responses following YFV-DakH1279 infection

**Figure 7:**
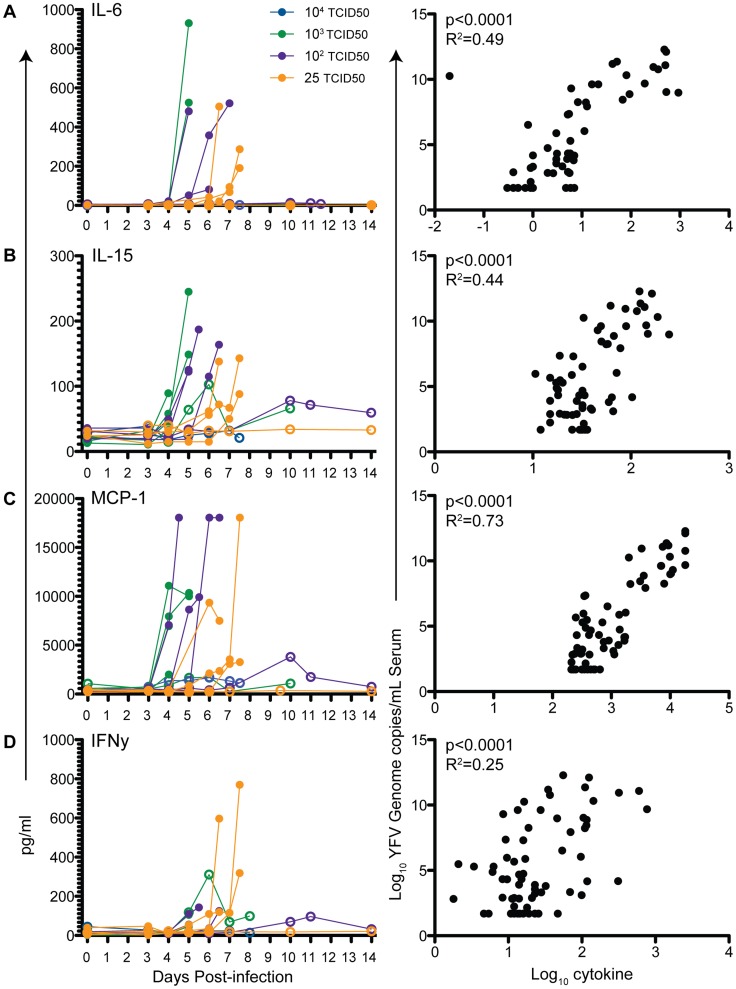
Circulating levels of inflammatory cytokines in YFV-DakH1279-infected rhesus macaques. Serum levels of IL-6 (A), IL-15 (B), MCP-1 (C), and IFNγ (D) increase sharply in animals requiring euthanasia following infection with YFV and significantly correlate with viral loads. Filled circles denote animals that required euthanasia and open circles denote animals that survived YFV infection.

We analyzed changes in serum cytokine levels associated with YFV-DakH1279 infection. Analysis of cytokines was affected by the heat inactivation step required to remove samples from the BSL-3 (see methods and materials). Levels of IL-4, IL-5, IL-8, IL-12/23p40, IL-17, G-CSF, GM-CSF, sCD40, and RANTES were either unchanged in post-infection samples or below levels of detection. In contrast, increased levels of IL-6, IL-15, MCP-1 and IFNγ were detected especially shortly before euthanasia. Levels of each cytokine/chemokine showed a significant correlation with viral load (p<0.001, with an R^2^ ranging from 0.25 for IFNγ to 0.68 for MCP-1) (Figure 7).

### YFV-DakH1279 and YFV-17D induce different transcriptomic responses

**Figure 8:**
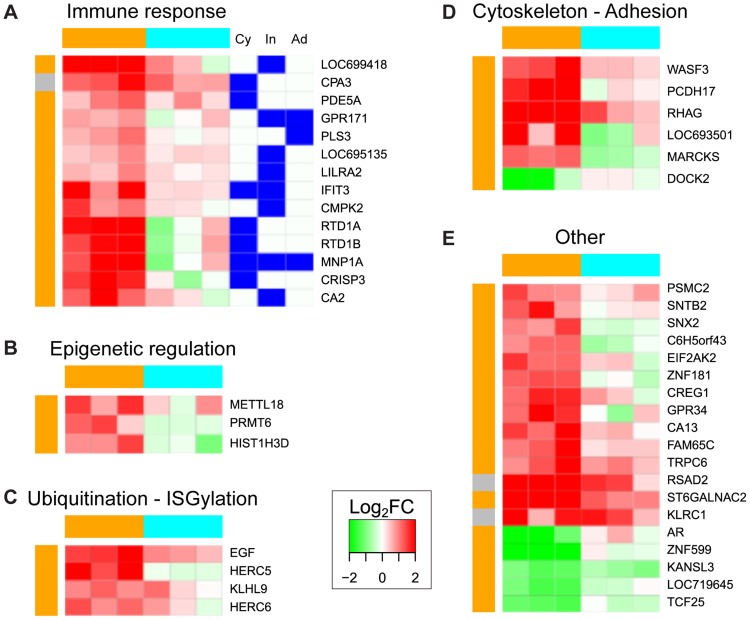
Functional characterization of PBMCs transcriptomic response to YFV-17D infection at 3 days post-infection. The 46 DEGs after YFV-17D infection were grouped into functional categories based on enriched gene ontology (GO) terms (Table S1) and their expression value in log_2_FC is depicted in a green to red gradient color scheme. Three animals were challenged with YFV-17D (orange), while another three animals were infected with YFV-DakH1279 (cyan). Color on the left of each heatmap indicates whether the gene was found differentially expressed after YFV-17D infection (orange), or both YFV-17D and YFV-DakH1279 (grey). Genes were functionally categorized into: (A) immune response category (these genes were either involved in cytokine signaling pathways (Cy), or specifically up-regulated in innate immune cells (In) or adaptive immune cells (Ad)); (B) epigenetics; 9C) Ubiquitination-ISGylation; (D) cytoskeleton-adhesion; or (E) did not map to a specific functional category.

To further explore the molecular basis of YFV pathogenesis, we performed gene expression profiling in PBMC isolated from three animals 0 dpi and 3 dpi with 10^3^ TCID_50_ of YFV-DakH1279 which required humane euthanasia (Figure 8). As a comparison, we included PBMC collected from three animals infected with one standard dose of the YFV-17D vaccine (6×10^4^ infectious unit) on 0 dpi and 3 dpi. PBMCs isolated from animals infected with 10^3^ YFV-DakH1279 were used because this dose elicits profound viscerotropic disease and the severe lymphopenia in animals infected with 5×10^4^ TCID50 made it difficult to obtain sufficient high quality RNA for microarray analysis from enough animals within this group. Day 3 was chosen because it preceded the severe lymphopenia observed in wild type YFV-DakH1279 infection (lymphocyte fold change 0 dpi and 3 dpi: 1.15, 0.85 and 0.94 respectively). No significant changes in lymphocyte numbers were observed following YFV-17D infection either (lymphocyte fold change 0 dpi and 3 dpi: 0.99, 1.14 and 1.18 respectively). Statistical analysis of gene profiles at 3 dpi compared to baseline levels (0 dpi) revealed that YFV-DakH1279 infection induced a more pronounced transcriptional response than YFV-17D infection. Specifically, 765 differentially expressed genes (DEGs) were detected following infection with YFV-DakH1279 (337 were downregulated and 428 genes were upregulated). In contrast, only 46 differentially expressed genes were identified following infection with YFV-17D (6 downregulated and 40 upregulated). Only 3 genes were shared between the two lists of DEGs: *KLRC1*, *CPA3* and *RSAD2*. All three genes, which are involved in the innate immune response to viral infection, were upregulated following YFV-17D or YFV- DakH1279 infection.

**Figure 9:**
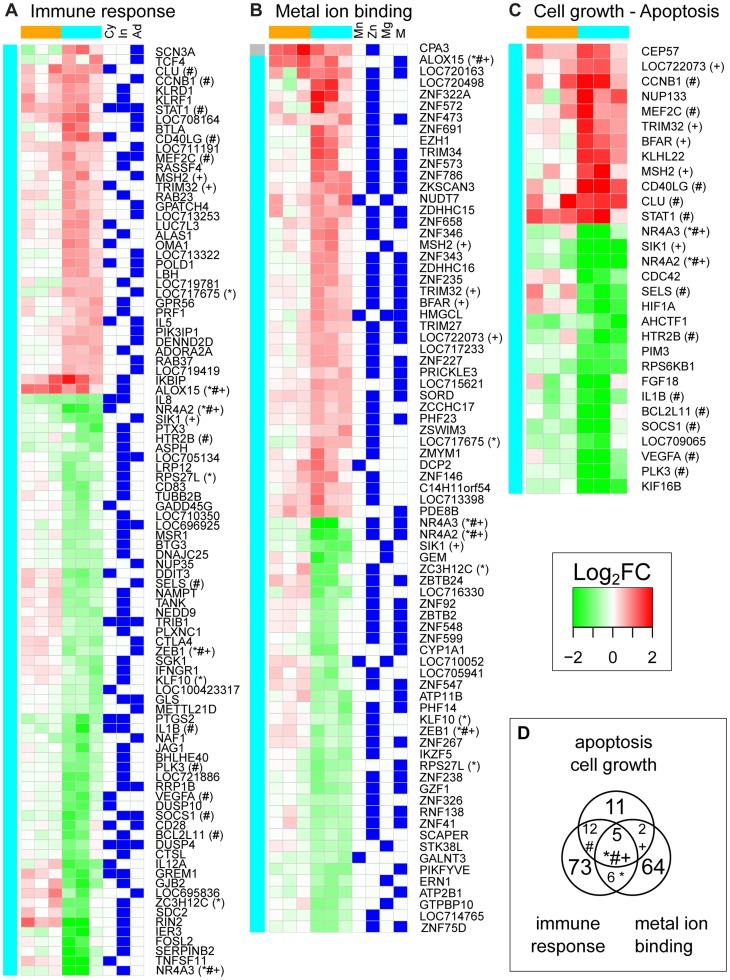
Functional characterization of PBMCs transcriptomic response to YFV-DakH1279 infection at 3 days post-infection. Expression values for DEGs after YFV-DakH1279 infection within the 3 main enriched functional categories (Table S1) are depicted in a green to red gradient color scheme. Three animals were challenged with YFV-17D (orange), while another three animals were infected with YFV-DakH1279 (cyan). Color on the left of each heatmap indicates whether the gene was found differentially expressed after YFV-DakH1279 infection (cyan), or both YFV-17D and YFV-DakH1279 (grey). Genes were functionally categorized into: (A) the immune response category (these genes were either involved in cytokine signaling pathways (Cy), or specifically up-regulated in innate immune cells (In) or adaptive immune cells (Ad)); (B) Metal ion binding (manganese ion binding (Mn), zinc ion binding (Zn) and other metal ion binding (M)); or (C) cell growth and apoptosis. (D) Extent of overlap between the three functional categories: # indicates genes common between cell growth/apoptosis and “immune response” pathways; + indicates genes common to “cell growth/apoptosis” and “metal ion binding” pathways; * indicates genes common to “metal ion binding” and “immune response” pathways.

We further characterized each transcriptional signature by performing functional enrichment [30]. This analysis revealed that DEGs after YFV-DakH1279 and YFV-17D infection belonged to different biological processes (Figure 8, 9). The only shared process between YFV-DakH1279 and YFV-17D was that of “immune response” (Figure 8A, 9A). However, while YFV-17D infection only induced up-regulation of immune response genes (Figure 8A), approximately two-thirds of the DEGs associated with immune response were down-regulated after YFV-DakH1279 infection (Figure 9A).

Genes specific to innate immune cells were enriched in both signatures (Enrichment Score (ES) = 4 for YFV-17D signature and 3.1 for YFV-DakH1279). Specifically, the 8 most highly upregulated genes following YFV-17D infection were associated with innate immune response to infection: *LOC699418* (eosinophil lysophospholipase-like), *RTD1A* (theta defensin 1 α precursor), *MNPA1* (α defensin 1 α), *CRISP-3* (cysteine-rich secretory protein 3), *IFIT3* (interferon-induced protein with tetratricopeptide repeats 3), *RSAD2* (radical S-adenosyl methionine domain containing 2-IFN induced gene) and *CPA3* (mast cell secreted carboxypeptidase A3). In contrast, 43 genes associated with innate immune cells were down-regulated following YFV-DakH1279 infection and only three genes were upregulated (*KLRC1*, *CPA3* and *RSAD2*). However, it should be noted that 35 genes associated with inflammatory responses, notably *STAT-1* (important for signaling through type I, II or III interferons), *IL-5*, and *CD40L* were upregulated following YFV-DakH1279 infection (Figure 9A).

With regards to the adaptive immune response, *PLS3*, also known as *T-plastin* was upregulated following YFV-17D infection. Expression of this gene is essential for germinal center formation and development of T-dependent antibody responses in mice [31], [32]. In contrast, numerous genes specific to B and/or T cells were dysregulated after YFV infection (ES = 2.1). For instance, *TNFSF11*, which is hypothesized to augment the ability of dendritic cells (DC) to stimulate naïve T cell proliferation, was downregulated [33]. Similarly, *SerpinB2*, believed to play a role in sculpting the adaptive immune response [34] is also downregulated and BTLA, a negative regulator of T and B cell responses is upregulated [35].

Functional categories enriched only after YFV-17D infection included ubiquitination and ISGylation, cytoskeleton and cell adhesion, and epigenetic regulation (Figure 8 B–D). Interestingly, among the 765 DEGs after YFV-DakH1279 infection, 115 had metal ion binding activity and over 80% of those genes are specifically involved in zinc ion binding (Figure 9B). An additional 30 genes were involved in cell growth or apoptosis regulation (Figure 9C) and 226 were related to transcription (GEO series accession number GSE51972). Finally, ingenuity pathway analysis revealed that the biological functions predicted to be the most activated after YFV-DakH1279 infection (based on gene ontology, literature and the Ingenuity knowledge database) were organismal death and cell death (z-score = 4.0 and 3.1), while the most inhibited was cell movement (z-score = −3.4). This analysis was based on the direction of change of the DEGs after infection. For example, CD28, a protein known to decrease apoptosis of T lymphocytes [36] and to increase migration of memory T cells [37] was down-regulated after YFV-DakH1279 infection whereas, RASSF4, a gene believed to be involved in apoptosis, was up-regulated [38]. Activation of pathways associated with cell death at 3 dpi could be involved in the lymphopenia observed during later stages of infection. Altogether, these results show that YFV-DakH1279 infection induce significant transcriptomic changes in PBMC at 3 dpi, before onset of symptoms and increase of blood biochemical markers of hepatic and kidney failure.

## Discussion

Yellow fever virus represents one of the most prevalent hemorrhagic fever viruses in the world today [39] and yet our understanding of YFV pathogenesis remains limited. In this study, we sought to address this gap in knowledge by characterizing yellow fever disease progression in rhesus macaques infected with the virulent strain, YFV-DakH1279. To further our understanding of the molecular basis of fatal versus non-fatal yellow fever disease, we also compared gene expression in PBMC collected on days 0 and 3 post-infection with YFV-DakH1279 or the attenuated vaccine strain, YFV-17D.

In 1928, Stokes and colleagues demonstrated that rhesus macaques were susceptible to the WT YFV Asibi strain and that disease can be readily transmitted from infected humans to rhesus macaques and from infected animals to naïve animals [40]. Those early studies played a critical role in the development of the currently used live attenuated vaccine [41]–[43]. Follow up studies by Dr. Bauer in 1931 showed that rhesus macaques were exquisitely sensitive to YFV and as little as 1 ml of inoculum containing 1∶10^9^ dilution of blood from an acutely infected animal resulted in disease transmission [44]. Several years later, Monath and colleagues also showed that YFV infection of rhesus macaques results in high viremia [21]. The data presented here provide an explanation for these earlier observations. Our studies show that acutely infected animals may harbor up to 10^12^ genome copies of YFV-DakH1279 per mL serum and that there is roughly a 1∶1 ratio between YFV genome copy number/mL serum and TCID_50_/ml [23]. It is therefore not surprising that lethal disease was induced in prior studies by administering 1∶10^9^ diluted blood as this may still contain up to 10^3^ TCID_50_ of virus. Indeed, in our studies, 75% of the animals infected with 25 TCID_50_ required humane euthanasia within 6 days of infection. Interestingly, two animals that survived at least 14 days after infection with 25 or 100 TCID_50_ presented with two successive rounds of viremia that occurred at 3–5 dpi and then again at 10–14 dpi. Viral loads in one of these animals even reached above 10^6^ genome copy number/mL serum during the second round of viremia. This is in agreement with the Bauer study that documented longer incubation periods (19 days in animals inoculated with small amounts of virus) [44]. However, in those studies, the disease was as severe as it was in animals that received larger doses of virus [44]. It is possible that the two animals in our study might have eventually showed more severe symptoms or succumbed to infection if they were not euthanized at the conclusion of the study on day 14. Additional studies are needed to determine whether inoculation with lower doses might result in a disease course that follows a longer time line similar to that observed in humans.

Monath and colleagues in 1981 [21] showed that yellow fever disease follows the same course in monkeys as described in humans but is more rapid and severe. In that study, seven rhesus macaques were infected subcutaneously with 1000 suckling mouse intracerebral LD_50_ of YFV-DakH1279. All of the animals required euthanasia by day 5 post-infection and showed high levels of viremia. Abnormalities in liver function tests were not detected until 24 hours before death and kidney dysfunction was only evident 18–12 hours before death. Similarly, data presented herein show that YFV infection in rhesus macaques results in severe viscerotropic disease even at very low inoculum doses with significant injury to liver and kidney also detected shortly before humane euthanasia. We detected Councilman bodies, (areas of hepatocyte degeneration) and Torres bodies (intranuclear eosinophilic granular inclusions) in postmortem liver samples. Moreover, evidence of tubular necrosis and protein deposits were seen in all kidney sections. These alterations are likely to lead to changes in renal hemodynamics and azotemia and eventually kidney failure. Similar to the study by Monath and colleagues [21], changes in levels of key indicators of liver and kidney function were often not evident until a few hours before the animals were humanely euthanized in our study.

In this study, we were able to assess the presence of viral antigen by IHC using a monoclonal antibody directed against YFV envelope [23]. This analysis revealed an unexpected finding. Although organ damage was evident in the liver, kidneys and lymphoid tissue, viral antigen was only detected in liver. These observations provide new insight into YF pathogenesis and suggest that tissue damage in the kidneys and lymphoid tissue may not be directly mediated by viral replication in situ, but more likely through soluble mediators that could potentially be produced elsewhere. Indeed, as described for fatal YF disease in humans [45]–[49], our analysis showed an increase in plasma levels of some cytokines shortly before euthanasia. These soluble mediators could be produced by the injured liver [46]–[49]. Another possibility is that some of these cytokines are secreted by injured endothelial cells as described for Ebola infection [50], a hemorrhagic fever that is also accompanied by profound lymphopenia [51]. It is also possible that these cytokines are produced by splenocytes. Additional studies are needed to address this question.

Interestingly, our transcriptomic analysis revealed that gene expression of several inflammatory cytokine genes such as *IL-8*, *IL-1β* and *IL-12* was down regulated in PBMC from YFV-DakH1279 infected animals. Although we did not measure the plasma protein levels of these specific cytokines in our luminex analysis, this outcome seems to contradict the increase in IFNγ, IL-15, IL-6 and MCP-1 plasma levels that we observed shortly before euthanasia. As discussed above, one possible explanation is that cytokines at the end stage of YF disease might not be produced by PBMC but rather by injured organs, notably the liver [46]. This hypothesis is supported by a recent study that showed robust pro- and anti-inflammatory cytokine gene expression and production by kupffer cells infected with YFV-Asibi [52]. High levels of circulating inflammatory factors may also result in the organ damage observed in the kidneys and lymphoid tissue in the absence of viral replication.

As previously described by Monath and colleagues [21], fatal YFV infection was accompanied by germinal center necrosis in secondary lymphoid tissues in our animals. In addition we also detected a corresponding dramatic loss of circulating lymphocytes. This lymphopenia preceded the appearance of clinical indicators of liver and kidney injury by ∼24 hours. Lymphopenia has also been observed in fatal cases of yellow fever vaccine-associated viscerotropic disease [9], [53], [54]. We also detected hemorrhagic foci in livers, red blood cells in kidneys, and congestion in spleens from animals that required euthanasia suggestive of a hemorrhagic disease. However, and in line with the earlier study by Monath and colleagues, changes in platelets and hematocrits were rather modest and only observed at endpoint euthanasia [21].

Using transcriptomic profiling, we found that many genes were dysregulated in lymphocytes at 3 dpi with YFV-DakH1279, including genes implicated in zinc binding and apoptosis (Figure 9C, D) that could contribute to lymphopenia. Our transcriptome data show that YFV-DakH1279 and YFV-17D induce vastly different host responses. Only 46 differentially expressed genes (DEGs) were detected after YFV-17D infection compared to 765 DEGs after YFV-DakH1279 infection. Since WBC counts were similar between the 2 groups of animals at 3 dpi, transcriptomic differences cannot be simply attributed to differences in cell composition but rather are more likely to reflect the direct impact of infection. The considerable differences in gene expression appear to correlate with the large differences in virulence and viral replication between these two viruses.

As described for humans [55], [56], our analysis shows that YFV-17D infection induces a robust innate immune response at day 3 post-infection in rhesus macaques. We also noted some interesting overlap between our gene list and the results published by Querec and colleagues [55] who examined human transcriptional responses following YFV-17D vaccination. Of note, expression of innate immune genes, *IFIT-3* and *RSAD2* (Figure 8A), as well as key transcription factor *EIF2AK2* (Figure 8E) and the E3 ubiquitin ligase *HERC5* (Figure 8C) were upregulated on day 3 in both studies.

Only three DEGs were found to be in common after infection with YFV-DakH1279 or YFV-17D and all three were upregulated innate immunity-related genes (Figure 8A, E; shaded grey). One of these genes, *RSAD2* (Figure 8E), encodes the anti-viral protein viperin (cig5). Viperin is multifunctional protein that is both IFN-dependently and independently induced in response to a number of diverse viral infections including several flaviviruses such as Hepatitis C virus, West Nile virus, and Dengue [57]. Increased expression of viperin was previously reported in HUVEC cells infected with either YFV-17D or wild type YFV-Asibi in vitro [58].

Another large portion of the DEGs detected after YFV-DakH1279 infection are related to metal ion binding and more specifically, to zinc ion binding. Interestingly, a transcriptome analysis also showed that zinc ion binding was among the most affected pathways in *Aedes aegypti* mosquitoes infected with different flaviviruses (West Nile virus, dengue virus and YFV) [59]. Zinc is an essential trace element in the human body that stimulates the activity of as many as 300 metal enzymes and metal-activated enzymes that are crucial for nucleic acid and protein metabolism [60]. Zinc deficiency causes various pathologic disorders including dysregulation of the immune response. For instance, zinc deficiency induces apoptosis in B cells and causes a decrease in absolute numbers [61]. Indeed, lymphopenia is one of the immunological hallmarks of zinc deficiency in humans and higher animals [62]. It is therefore possible that dysregulation in zinc binding pathways could explain the severe lymphopenia that we observed in YFV infected animals as viremia increases. Interestingly, lower circulating zinc levels have been noted during Hepatitis C virus infection [63], [64]. Future studies should investigate changes in circulating zinc levels during YFV infection as well as the mechanisms underlying changes in zinc levels during flavivirus infection and their utility as prognostic indicators of disease severity.

In summary, the study described here indicates that wild type YFV-DakH1279 infection leads to severe lymphopenia and rapid multi-organ failure of adult rhesus macaques. This outstanding model for studying human YF infection can be confidently expanded for evaluating novel vaccines and therapeutics. The lymphopenia precedes changes in key indicators of liver and kidney injury and may provide an earlier clinical outcome measure of subsequent disease severity. Another novel key observation in our study is that YFV appears to have replicated almost exclusively in the liver and thus additional organ damage is most likely due to soluble mediators, potentially secreted by the liver. Our data also show a first look at robust alteration of the host transcriptional program following infection with wild type YFV-DakH1279 with induction of pathways associated with apoptosis and dysregulation of immune response genes including down regulation of innate immune response, inhibition of lymphocyte trafficking, disruption of ion (and more specifically zinc) binding and increased apoptosis. Further studies will be important to characterize the potential role of ion binding and immune gene dysregulation in lymphopenia and disease outcome.

## Supporting Information

Figure S1 **Hematological indicators following YFV-DakH1279 infection in rhesus macaques.** Percentage hematocrit (A), hemoglobin concentration (B), and platelet numbers (C) were determined using Hemavet instrument at the indicated time points post infection. Filled circles denote animals that required euthanasia and open circles denote animals that survived YFV infection.
(TIF)

Figure S2 **YFV-DakH1279 infection results in a selective loss of peripheral B and T cells in rhesus macaques.** Frequencies of CD4+ (A) and CD8+ (B) T cells, CD20+ B cells (C), and lineage negative HLA-DR+CD14+ monocytes (D) were determined using flow cytometry at the indicated time points after infection. Filled circles denote animals that required euthanasia and open circles denote animals that survived YFV infection.
(TIF)

Table S1 **Summary of functional enrichment categories for differentially expressed genes.** GO = gene ontology; 17d = 17D vaccine; yfv = YFV-DakH1279; ES = enrichment score; category = functional category.
(XLSX)
