# Supplementary figures and images for: Pathophysiologic and Transcriptomic Analyses of Viscerotropic Yellow Fever in a Rhesus Macaque Model
Source: PLoS Negl Trop Dis. 2014 Nov 20;8(11):e3295. doi: 10.1371/journal.pntd.0003295 (PMC4238990; doi:10.1371/journal.pntd.0003295)

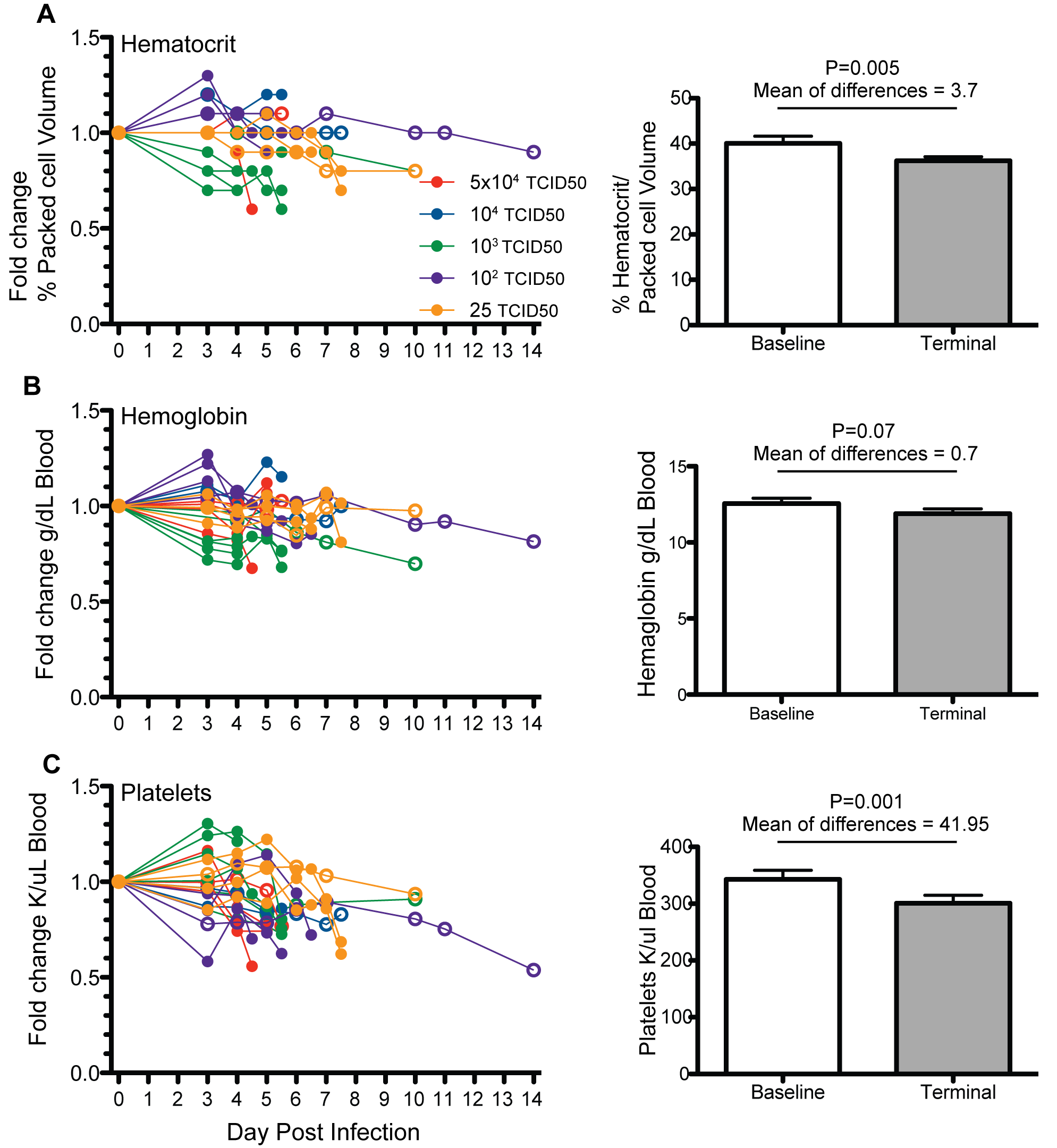

Supplement: Figure S1 — Hematological indicators following YFV-DakH1279 infection in rhesus macaques. Percentage hematocrit (A), hemoglobin concentration (B), and platelet numbers (C) were determined using Hemavet instrument at the indicated time points post infection. Filled circles denote animals that required euthanasia and open circles denote animals that survived YFV infection. (TIF) [file pntd.0003295.s001.tif]

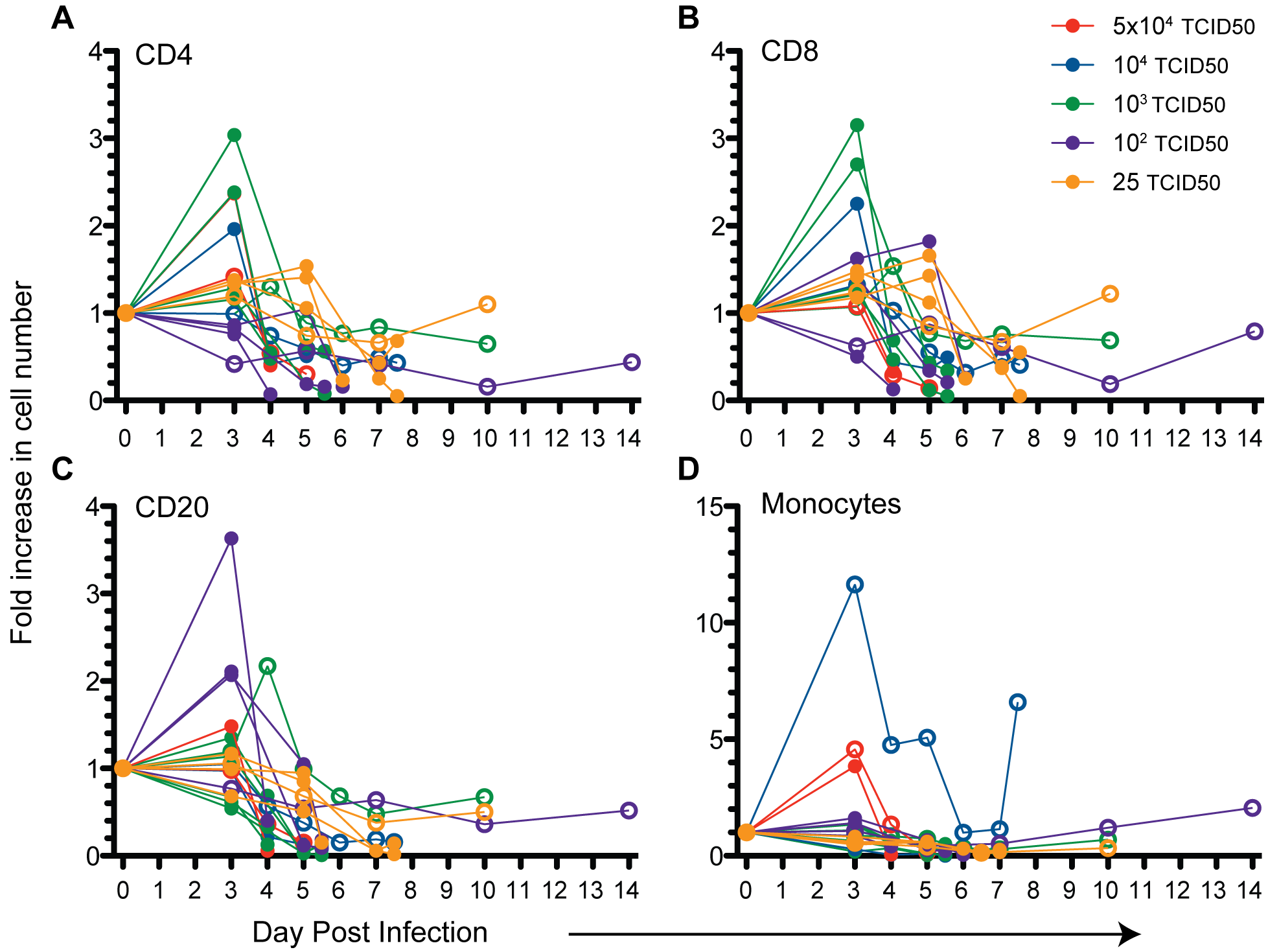

Supplement: Figure S2 — YFV-DakH1279 infection results in a selective loss of peripheral B and T cells in rhesus macaques. Frequencies of CD4+ (A) and CD8+ (B) T cells, CD20+ B cells (C), and lineage negative HLA-DR+CD14+ monocytes (D) were determined using flow cytometry at the indicated time points after infection. Filled circles denote animals that required euthanasia and open circles denote animals that survived YFV infection. (TIF) [file pntd.0003295.s002.tif]
